# Eosinophilic angiitis presenting with a true, fusiform, temporal artery aneurysm

**DOI:** 10.1002/ccr3.869

**Published:** 2017-04-08

**Authors:** Megan MacDiarmid, Dmitri Nepogodiev, Alok Tiwari, Martin Duddy, Martyn Carey, Paresh Jobanputra

**Affiliations:** ^1^ Department of RheumatologyQueen Elizabeth Hospital BirminghamMendelsohn Way, EdgbastonBirminghamB15 2THUK; ^2^ Department of Vascular SurgeryQueen Elizabeth Hospital BirminghamMendelsohn Way, EdgbastonBirminghamB15 2THUK; ^3^ Department of Interventional RadiologyQueen Elizabeth Hospital BirminghamMendelsohn Way, EdgbastonBirminghamB15 2THUK; ^4^ Department of PathologyQueen Elizabeth Hospital BirminghamMendelsohn Way, EdgbastonBirminghamB15 2THUK

**Keywords:** Digital ischemia, eosinophilic polyangiitis

## Abstract

Temporal artery aneurysm is a rare cause of temporal artery swelling in the absence of preceding trauma. Vasculitis other than giant cell arteritis, such as eosinophilic granulomatosis with polyangiitis, should be considered in such cases and a careful assessment of other medium‐sized arteries undertaken.

## Introduction

Superficial temporal artery aneurysms are rare and are largely reported in the literature with case studies alone 1. Those reported are largely pseudoaneurysms, where there is a break in the arterial wall secondary to trauma, and true aneurysms, where all three layers of the arterial wall are intact, are exceptionally rare 2. Around 85% of temporal artery aneurysms follow trauma 3. Both penetrating and blunt head injuries are implicated 4. In either case, the vessel wall is either partially divided or suffers a contusion, leading to necrosis. Vessel wall inflammation in vasculitis can lead to weakness of all three vessel wall layers and subsequent true aneurysm formation. This is a recognized complication of medium‐vessel vasculitides 5.

Differential diagnoses for temporal masses include lipomata, sebaceous cysts, dermoid cysts 6, arteriovenous fistulae, and arterial tumors 4.

## Case History

A 51‐year‐old man was referred urgently to our vascular clinic with a five‐week history of an enlarging lump in the left temporal area. He felt the swelling had developed after forceful nose blowing during an upper respiratory illness a few weeks before presentation. He had lost around 3 kg in weight. There was no history of trauma. He complained of an occipital headache but had no other symptoms. He gave a history of rhinitis and had used a corticosteroid inhaler in the past. His sister had lupus, his mother rheumatoid arthritis and his father cerebrovascular disease. He had ceased smoking a year previously but had a 15 pack‐year history.

A blood count was normal other than a raised eosinophil count (1.8 × 10^9^/L). Blood chemistry including immunoglobulins, renal and liver function, and C‐reactive protein (CRP) was normal. His erythrocyte sedimentation rate (ESR) was 15 mm/h. Other tests including antineutrophil cytoplasmic antibody (ANCA), antinuclear antibody (ANA), anti‐DNA, antiextractable nuclear antigen (ENA) antibodies, and serology for hepatitis B, C and human immunodeficiency virus (HIV) were negative.

A duplex ultrasound scan of the left temporal artery showed a 4‐cm‐long fusiform aneurysm. A small amount of extramural thrombus was seen in the dilated segment. There was no perivascular edema (halo sign) in either temporal artery. Histology of the resected aneurysm revealed pan‐arteritis consisting of lymphocytes and eosinophils, most marked in the intima. There was intimal hyperplasia showing marked focal vascular proliferation. No giant cells were present. A systemic vasculitis was thought possible, but it was believed that the changes most resembled juvenile temporal arteritis ‐– non‐giant‐cell granulomatous inflammation of the temporal arteries found in children and young adults 6. This condition typically presents as a temporal artery aneurysm but is otherwise asymptomatic. It is not associated with systemic disease, resolves with resection and is felt to have a good prognosis 3.

In our rheumatology clinic, a recent history of white finger was noted. He had developed painful finger tips with reduced sensation. The left radial artery was thought to be ectatic and the left brachial pulse more forceful. No arterial bruits were heard. A further swelling, adjacent to the resected temporal artery, was found. A further duplex ultrasound scan showed left temporal artery enlargement distal to the resected artery, widening of the left distal radial artery, an irregular lumen of the left ulnar artery, and obliteration of the lumen of the right ulnar artery. Blood test results for thrombotic disorders were negative. Magnetic resonance angiography demonstrated occlusion of the left radial artery at the wrist. The right radial artery was patent and in good condition.

**Figure 1:**
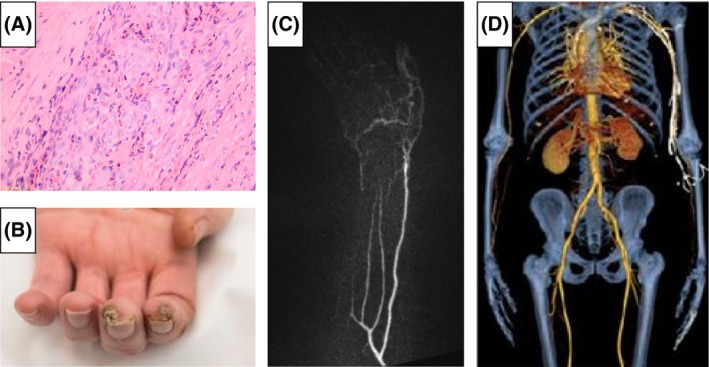
(A) Histology from temporal artery aneurysm demonstrating eosinophilia. (B) Photograph of left hand showing ischemic digits. (C) Left arm magnetic resonance angiography (MRA) scan demonstrating an abrupt occlusion of the left radial artery at the level of the wrist. (D) Computed tomography angiogram (CTA) scan demonstrating no other vascular lesions elsewhere in the body.

Eosinophilic polyangiitis was diagnosed. He was treated with prednisolone 40 mg per day, aspirin, amlodipine, sildenafil, and prostacyclin infusions for digital ischemia. Despite this, there was increasing pain, ulceration, and tissue loss of the tips of his index and middle fingers (Fig. 1). Intravenous cyclophosphamide 500 mg every 2 weeks on three occasions and every 3 weeks on a further three occasion was given.

## Outcome

In time, the ischemia in his right hand improved substantially but symptoms persisted in the left hand, affecting the tip of his left middle finger. Repeat duplex ultrasound scan, 8 months after presentation, showed return of flow to both ulnar arteries and that thickening of the ulnar arteries had reduced. Fifteen months after presentation, he remains in full employment while taking prednisolone 5 mg, azathioprine 125 mg per day, sildenafil, aspirin 75 mg, amlodipine, and atorvastatin. The tips of the left index and middle fingers remain cool, but the right hand is well perfused and there is no swelling of his temporal artery.

## Discussion

True aneurysms of the temporal artery, as opposed to pseudoaneuysms caused by trauma, are exceptionally rare. Differential diagnoses include systemic vasculitis such as giant cell arteritis (GCA), although temporal artery aneurysms due to GCA are also exceptionally rare. Most temporal artery aneurysms are thought to be benign and require little more than simple resection 1. A Valsalva maneuver, such as one which might occur with forceful nose blowing, may rarely cause a temporal artery pseudoaneuysm 7. Our patient had a true aneurysm with confirmed arteritis. It was unclear whether forceful nose blowing was relevant.

Eosinophilic granulomatosis with polyangiitis (EGPA, previously Churg–Strauss syndrome) is a systemic necrotising vasculitis affecting small and medium‐sized blood vessels 8. Although classed as an ANCA‐associated vasculitis, ANCA is nevertheless only positive in around a third of affected individuals 9 and ANCA negativity does not rule out the diagnosis. It is a rare disorder and less prevalent than the other smaller vessel vasculitides. Prevalence is estimated to be 7–13 per 1,000,000 in a multiethnic population 10.

The classification criteria for EGPA, as defined by the American College of Rheumatology, include asthma, eosinophilia (>10% on differential white blood cell count), and evidence of end‐organ damage, in particular of the upper or lower respiratory tract, the vasculature, or peripheral nerves 11.

While acute phase reactants are usually raised in acute vasculitis, this is not a uniform finding and ESR and CRP levels should not be used to monitor disease activity 12. The five‐factor score' (one point each for renal insufficiency, proteinuria, gastrointestinal involvement, central nervous system involvement, and cardiomyopathy) has been shown to predict five‐year survival in EGPA, polyarteritis nodosa (PAN), and microscopic polyangiitis (MPA) 13.

Guidelines for the management of ANCA‐associated vasculitis 14 divide treatment into an induction phase and a maintenance phase. Life‐ or organ‐threatening disease should be suspected in all cases and, where there is evidence, induction with high‐dose glucocorticoids and pulsed intravenous cyclophosphamide or rituximab is recommended.

Cyclophosphamide should be given as intravenous pulses every 2 weeks for three doses, followed by every 3 weeks. Total duration of treatment should be 3–6 months and until remission is achieved. This regimen has been shown to be as effective in inducing remission as long‐term low‐dose oral treatment 15 but with a lower cumulative dose, thus reducing the risk of toxicity including leucopenia and hemorrhagic cystitis 16. Pulsed regimens have, however, been shown to carry a higher risk of relapse 17. It is generally accepted that cumulative lifetime dose of cyclophosphamide should not exceed 25 g.

The standard intravenous dose of cyclophosphamide is 15 mg/kg, up to a maximum of 1500 mg. However, low‐dose regimens, with a fixed dose of 500 mg regardless of weight, followed by maintenance with azathioprine have been shown to produce good long‐term results measured by mortality, renal function, and proteinuria 18, and to reduce drug‐related adverse events without affecting remission rates in older patients 19. A low‐dose regimen was used in this case to good effect.

Randomized trials have demonstrated that rituximab is noninferior to cyclophosphamide for inducing remission 20. The recommended dose is 375 mg/m^2^/week for 4 weeks 14. It is primarily indicated in younger patients to avoid infertility. It is also suggested in patients where there is a high risk of infection, although trial data have not demonstrated lower rates of serious infections as anticipated.

Where there is no evidence of organ damage, as in this case, induction with high‐dose glucocorticoids and methotrexate should be considered. Corticosteroid monotherapy may be sufficient in a majority of similar patients, but around a third may relapse and need additional immunosuppressive therapy 21. Normal inflammatory markers and the absence of renal and other systemic disease, giving a ‘five‐factor score’ of zero, suggested that our patient had a good prognosis. Thus, corticosteroids alone may have sufficed. Our patient, however, developed digital ischemia with tissue damage, necessitating the use of cyclophosphamide.

We feel that our patient has a rare presentation of eosinophilic granulomatosis with polyangiitis (EGPA or Churg‐Strauss) based on histology, eosinophilia, and rhinitis, despite the absence of asthma. Asthma may develop after vasculitis 22, although typically the vasculitic phase of EGPA develops after asthma and eosinophilia 23. Our patient had a recurrence of rhinitis on steroid taper but has not developed asthma so far. In addition, the absence of a significant acute‐phase response has led to a dependence on imaging to aid clinical judgment when adjusting therapy or monitoring progress. Our patient has maintained a normal acute phase response throughout his illness. Low‐grade eosinophilia, maximum 0.8 × 10^9^/L, recurred on steroid taper without clinical relapse, other than short‐lived symptoms of rhinitis.

In conclusion, swellings over the temple may be due to temporal artery aneurysms. Most such swellings are pseudoaneurysms secondary to trauma. Rarely, as in this case, aneurysms may be due to vasculitis. While aneurysms are a recognized complication of medium‐ and large‐vessel vasculitis, it is important to remember that they may develop in rare sites. Vigilance and consideration of vasculitis, other than giant cell arteritis, are needed when faced with temporal artery disease including an aneurysm.

## Consent

Written informed consent was obtained from the patient for publication of this case report and any accompanying images. A copy of the written informed consent is available for review by the Editor of this journal.

## Authorship

MM: assessed the patient in clinic, and drafted and revised the manuscript. DN: assessed the patient in clinic, and drafted the manuscript. AT: drafted and revised the manuscript. MD: reported performed duplex scans of the temporal artery and upper limbs and reported MRA scan of the upper limbs and revised the manuscript. MC: reported the histological findings and revised the manuscript. PJ: assessed and managed the patient and drafted and revised the manuscript.

## Conflict of Interest

The authors have no competing interests to declare.
